# Childhood Hospitalisation with Infection and Cardiovascular Disease in Early-Mid Adulthood: A Longitudinal Population-Based Study

**DOI:** 10.1371/journal.pone.0125342

**Published:** 2015-05-04

**Authors:** David P. Burgner, Matthew N. Cooper, Hannah C. Moore, Fiona J. Stanley, Peter L. Thompson, Nicholas H. de Klerk, Kim W. Carter

**Affiliations:** 1 Murdoch Childrens Research Institute, Royal Children’s Hospital, Parkville, Victoria, Australia; 2 School of Paediatrics and Child Health, University of Western Australia, Crawley, Western Australia, Australia; 3 Department of Paediatrics, University of Melbourne, Parkville, Victoria, Australia; 4 Department of Paediatrics, Monash University, Clayton, Victoria, Australia; 5 Division of Population Sciences, Telethon Kids Institute, University of Western Australia, Subiaco, Western Australia, Australia; 6 Department of Cardiovascular Medicine, Sir Charles Gairdner Hospital, Nedlands, Western Australia, Australia; 7 School of Medicine and Pharmacology and Population Health, University of Western Australia, Crawley, Western Australia, Australia; 8 McCusker Charitable Foundation Bioinformatics Centre, Telethon Kids Institute, University of Western Australia, Subiaco, Western Australia, Australia; Temple University School of Medicine, UNITED STATES

## Abstract

**Background:**

Pathogen-specific and overall infection burden may contribute to atherosclerosis and cardiovascular disease (CVD), but the effect of infection severity and timing is unknown. We investigated whether childhood infection-related hospitalisation (IRH, a marker of severity) was associated with subsequent adult CVD hospitalisation.

**Methods:**

Using longitudinal population-based statutorily-collected administrative health data from Western Australia (1970-2009), we identified adults hospitalised with CVD (ischaemic heart disease, ischaemic stroke, and peripheral vascular disease) and matched them (10:1) to population controls. We used Cox regression to assess relationships between number and type of childhood IRH and adulthood CVD hospitalisation, adjusting for sex, age, Indigenous status, socioeconomic status, and birth weight.

**Results:**

631 subjects with CVD-related hospitalisation in adulthood (≥ 18 years) were matched with 6310 controls. One or more childhood (< 18 years) IRH was predictive of adult CVD-related hospitalisation (adjusted hazard ratio, 1.3; 95% CI 1.1-1.6; *P* < 0.001). The association showed a dose-response; ≥ 3 childhood IRH was associated with a 2.2 times increased risk of CVD-related hospitalisation in adulthood (adjusted hazard ratio, 2.2; 95% CI 1.7-2.9; *P* < 0.001). The association was observed across all clinical diagnostic groups of infection (upper respiratory tract infection, lower respiratory tract infection, infectious gastroenteritis, urinary tract infection, skin and soft tissue infection, and other viral infection), and individually with CVD diagnostic categories (ischaemic heart disease, ischaemic stroke and peripheral vascular disease).

**Conclusions:**

Severe childhood infection is associated with CVD hospitalisations in adulthood in a dose-dependent manner, independent of population-level risk factors.

## Introduction

Cardiovascular disease (CVD) is the leading cause of mortality worldwide.[1] The underlying pathology, atherosclerosis, is a chronic inflammatory process beginning in early life, with a long asymptomatic phase. The extent of low grade inflammation in atherosclerosis is strongly predictive of CVD outcomes.[2] The contribution of infection to chronic vascular inflammation, atherosclerosis and CVD is controversial. Studies have sought to implicate specific microbes (particularly *Chlamydophilia pneumoniae*, *Helicobacter pylori*, periodontal pathogens, and chronic viral infections),[3] or the overall infectious burden,[4]; there are data to support both pathogen-specific and non-specific associations. Serological evidence of exposure to multiple pathogens correlates with endothelial dysfunction, coronary artery atherosclerosis and increased inflammatory mediators,[5] independent of traditional CVD risk factors.[6] However cross-sectional serological studies are only informative for a fraction of the overall infection burden and are not indicative of the severity or timing of infection.

To investigate the relationship between severe childhood infection and adult CVD, we analysed unique longitudinal population-based data for infection-related hospitalisation (IRH, a marker of severity) and adult CVD-related hospitalisation, in the same individuals over 40 years.

## Methods

### Western Australian Data Linkage System

The Western Australian Data Linkage System (http://www.datalinkage-wa.org.au) is a unique total population-based resource of links between administrative data on over 3.8 million individuals who have lived in Western Australia (current total population ~2.5 million) over the last four decades.[7] It links statutory data collections, including all births, deaths, marriage registrations, electoral roll registrations, hospitalisations, mental health service contacts, emergency department presentations, midwife notifications, and cancer registrations. In the current analyses, we used data from the Hospital Morbidity Database System, the Midwives' Notification System, the Birth Register, and the Death Register. The Hospital Morbidity Database System has 100% coverage of private and public hospital admissions throughout Western Australia,[7] and the Midwives’ Notification System has validated identification of Indigenous status. The study was approved by the University of Western Australia Human Research Ethics Committee and the Data Linkage Unit of the Department of Health of Western Australia. No individual consents were obtained (in keeping with the ethics committee approvals) as the data were anonymised before being shared with the researchers. Analysis was performed on de-identified population-level data.

### Subject selection

We conducted a case-control study using data covering the period January 1970 (the initiation of electronic hospital records in Western Australia) to December 2009. Coding of hospital diagnoses was based on the International Classification of Diseases, 8^th^ Revision (ICD8) (1970–1978), 9^th^ Revision (ICD9) (1979–1987), ICD9 Clinical Modification (ICD9CM) 1988–1999), and the International Statistical Classification of Diseases and Related Health Problems, 10^th^ Revision Australian Modification (ICD10AM) from July 1999 onwards.

Cases were Western Australian-born individuals (born from 1970 onwards), hospitalised within Western Australia after their 18^th^ birthday with a CVD diagnosis (pre-defined as angina, ischaemic heart disease, ischaemic—but not haemorrhagic—stroke, or peripheral vascular disease, S1 Table). We matched cases at a 1 to 10 ratio by sex, and by month and year of birth to individuals born in Western Australia selected from the Western Australian Data Linkage System who had no recorded CVD hospitalisation. This case to control ratio allowed for analysis of the low prevalence of childhood infection-related hospitalisation (IRH) within pre-defined clinical categories of infection (S2 Table).

### Diagnostic coding of infection

We identified all IRH before 18 years of age in cases and controls for which there was at least one pre-defined infection-related diagnostic code (S2 Table). We grouped IRH *a priori* into six common clinical categories (upper respiratory tract infection, lower respiratory tract infection, infectious gastroenteritis, urinary tract infection, skin and soft tissue infection, and other viral infection), based on the Clinical Classification Software and previous analyses (S2 Table).[8,9] Individual IRH with diagnoses within more than one diagnostic infection group were excluded from the diagnostic subgroup analyses, as it was not possible to determine the clinically most significant infection for a given IRH. We excluded individuals whose IRH included rheumatic fever, which has an unusually high incidence in the Australian Indigenous population,[10] as these data may be less relevant to other settings.

Childhood infections not resulting in hospitalisation are not captured by statutory hospital-based morbidity data. In an attempt to investigate recurrent and chronic infections, which usually do not necessitate hospitalisation, we also analysed childhood hospitalisations with operative procedure codes relating to chronic or recurrent middle ear infections (otitis media), including ventilation tube insertion and repair of tympanic membrane perforation (S4 Table).

### Other possible determinants of CVD-related hospitalisation

Indigenous status was defined by self-report in 75% or more of an individual’s available records across the accessed datasets, using national best practice guidelines for data linkage coding relating to Indigenous status.[11] Data on birth weight, gestational age and birth characteristics were available on a subset of the subjects (1980 onwards) through the Midwives’ Notification System. We used a measure of birth weight contingent on gestational age, termed ‘percentage of optimal birth weight’ (POBW), which represents the ratio of observed birth weight to ‘optimal’ birth weight, derived from a total population of singleton births, adjusted for gestational age, maternal height, parity and infant sex.[12] We utilised the Socio-Economic Index for Areas,[13] a measure of socio-economic status derived by the Australian Bureau of Statistics from national census data, as a robust measure of relative socioeconomic disadvantage. This reflects the general level of education and occupation-related skills of individuals within a census collection district (approximately 250 households within an urban area) and includes measures of education qualifications, occupation, and unemployment. This index is highly correlated with other Australian Bureau of Statistics measures of disadvantage and does not include Indigenous status in the calculation. We used the state-level cut-points of the Australian Bureau of Statistics Socio-Economic Index for Areas (based on the 1996 national census, the most complete available dataset) for the Index of Education and Occupation measure to define the relative social disadvantage of the population, based on an individual’s collection district at birth, as follows: <10% of the population (least disadvantaged), 10%-25%, 25%-50%, 50%-75%, 75%-90%, and >90% (most disadvantaged).

To assess whether childhood hospitalisation *per se* (irrespective of whether it was due to infection) may partly reflect social disadvantage, we also analysed the relationship between trauma-related hospitalisations in childhood (which show social gradients [14]) and CVD-related admissions in adulthood. Trauma-related hospitalisations in those < 18 years of age were categorised using pre-defined ICD codes from CCS groupings. We could therefore partly adjust for social disadvantage in the whole study group.

### Statistical analyses

Cox proportional hazard models were used to calculate hazard ratios for which the outcome was CVD-related hospitalisation (≥ 18 years of age), and the time to event was the number of months until the first CVD-related hospitalisation, the date of death, or end of data collection (December 2009), whichever occurred first. Adjusted hazard ratios (aHR) and 95% confidence intervals (95% CI) for all variables of interest were calculated from multivariate models. Analyses were adjusted for age, sex and Indigenous status, parameters associated with increased risk of CVD and of childhood IRH.[8,15] Separate analyses were performed for non-Indigenous and Indigenous subsets. Regression models were adjusted for birth weight and social disadvantage where data were available. The proportional hazards assumption was assessed visually using Schoenfeld residuals plots and confirmed by testing time-dependent interaction variables. Infection-related hospitalisations were analysed as a dichotomous variable (none or any IRH), by the number of hospitalisations (0, 1, 2 and ≥ 3 IRH), and the number of hospitalisations for each of the clinical infection group (0, 1 and ≥ 2 admissions). All data manipulation and analysis was performed in R version 3.

Cox regression was used because of the strong dependency of CVD on age. However, because of the over-representation of cases in a case-control study design, where all cases but only a proportion of controls are included, effect estimates may be: (i) biased downward if cases are included from the start of follow-up (before their first episode of case-defining CVD-related hospitalisation), or (ii) biased upward if cases are only included once the CVD-related hospitalisation has occurred.[16] If these differences are large, then a correction method that applies population-based weights to the controls may be used to give unbiased estimates.[17] Differences between the two sets of estimates were investigated in the current datasets, so that these corrections could be applied if necessary.

## Results

### Characteristics of the study population

631 subjects had at least one CVD-related hospitalisation in adulthood and were defined as cases. They were frequency-matched by age (month and year of birth) and sex to 6310 controls. The mean age of first CVD admission was 29.8 years. Males represented 64% of the study population, and 207 (32.8%) cases were Indigenous, compared with 526 (8.3%) of controls (Table 1).

**Table 1:** Characteristics of Subjects.

*(a)* All Subjects		
	Mean (SD)	
	Cases (n = 631)	Controls (n = 6310)
**Age of onset (years)** ^**1**^	29.8 (5.5)	-
**Maximum age (years)** ^**2**^	34.5 (4.8)	35.0 (4.4)
	*% [n]*	
**Sex**		
Male	63.6 [401]	63.6 [4010]
Female	36.4 [230]	36.4 [2300]
**Indigenous status**		
Non-Indigenous	67.2 [424]	91.7 [5784]
Indigenous	32.8 [207]	8.3 [526]
*(b)* Subjects with birth and socioeconomic data available		
	**Cases (n = 68)**	**Controls (n = 738)**
	*Mean (SD)*	
**Age of onset (years)** ^**2**^	23.0 (2.7)	-
**Proportion of Optimal Birth Weight (%)** ^**3**^	98.2 (15.7)	97.5 (12.6)
**Maximum age (years)** ^**2**^	25.6 (2.8)	26.2 (2.7)
	*% [n]*	
**Sex**		
Male	57.4 [39]	59.9 [442]
Female	42.6 [29]	40.1 [296]
**Indigenous status**		
Non-indigenous	64.7 [44]	93.6 [691]
Indigenous	35.3 [24]	6.4 [47]
**Social disadvantage** ^**4**^		
1 (low)	5.3 [3]	8.0 [49]
2	12.3 [7]	14.7 [90]
3	29.8 [17]	24.5 [150]
4	19.3 [11]	23.5 [144]
5	15.8 [9]	17.5 [107]
6 (high)	17.5 [10]	11.8 [72]

^1^ Age of onset derived from the difference, in years, between data of first CVD event and date of birth

^2^ Maximum age is the age reached by the end of the study period, December 2009

^3^ POBW data was only available for a subset of the data, births after 1980

^4^ Social disadvantage was based on sextiles of the 1996 Australia Bureau of Statistics Index of Education and Occupation

Percentage of optimal birth weight (POBW) data were available for 806 individuals from the study population, 68 (10.8%) cases and 738 (11.7%) controls, with the mean age of cases with POBW slightly younger relative to all cases, reflecting the fact that birth data were available more recently (1980 onwards) than the whole cohort (1970 onwards). In those with POBW data, the proportion of males and Indigenous individuals were similar to the overall cohort, and did not differ significantly between cases and controls (Table 1). Socioeconomic status data were available in the same subset of 804 individuals and the overall distribution of socioeconomic status was similar between groups; 47.4% of cases and 47.2% of controls had standardised deprivation scores in the lowest three categories (Table 1).

### Associations between childhood IRH and adult CVD

**Fig 1:**
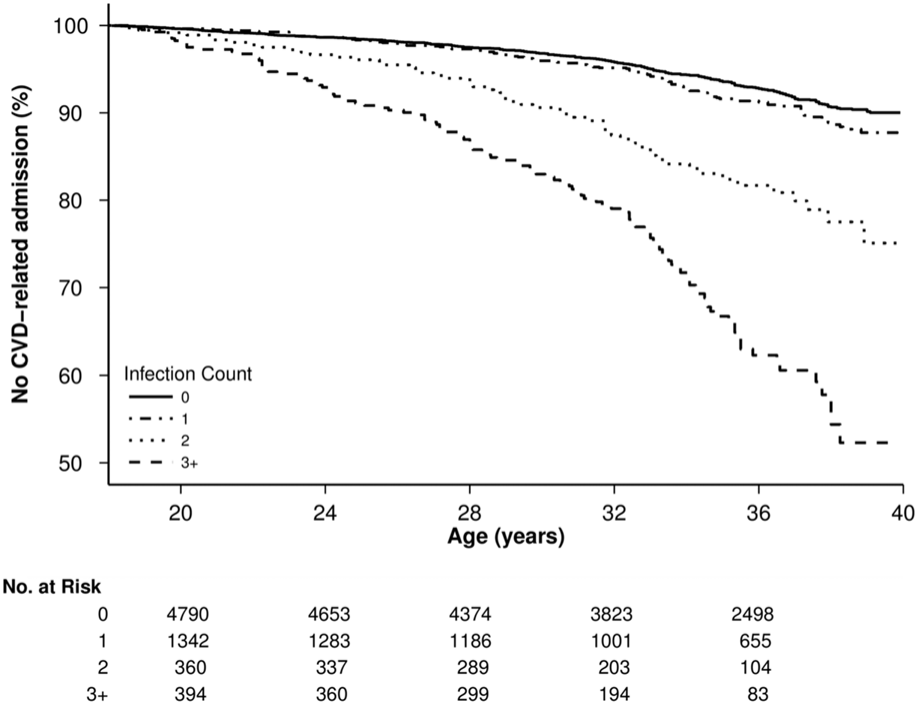
Kaplan-Meier plot showing survival (no CVD event in adulthood) and number of subjects at risk by age for increasing counts of hospitalisation for any infection in childhood.

Hospitalisation at least once with an infection during childhood was significantly associated with adult CVD-related hospitalisation, after adjustment for sex, year of birth and Indigenous status (aHR, 1.3; 95% CI 1.1–1.6, *P* < 0.001). Infection-related hospitalisations in childhood showed a dose-response association with subsequent CVD-related hospitalisations; 115 cases (18.2%) had three or more childhood infectious disease hospitalisations, compared with 288 (4.6%); aHR, 2.2; 95% CI 1.1–2.9; *P* < 0.001 (Table 2—Model a, Fig 1). In those with birth weight data, the relationship between childhood infection-related hospitalisations and adult CVD was of the same magnitude; 24 of 68 (35.3%) cases had 3 or more infectious disease admissions compared with 74 of 738 (10.0%) controls; aHR, 2.3; 95% CI 1.1–5.0; *P* = 0.04, after adjustment for year of birth, sex, Indigenous status, POBW and social disadvantage (S3 Table). Stratified analyses for ≥ 2 IRH (to ensure sufficient case events) in non-Indigenous individuals only were consistent with estimates for the overall study population (aHR 1.8; 95% CI 1.3–2.4; *P* < 0.001), and similarly in Indigenous individuals only (aHR 2.25; 95% CI 1.6–3.2; *P* < 0.001). Analyses included all eligible person-time from the cases, so analyses were repeated with cases only included at the time of their admission, as described in the Methods. As estimates of aHR only changed in the second or third decimal place, correction by re-weighted analysis was unnecessary.

**Table 2:** Adjusted Hazard Ratios for CVD Hospitalisation in Adulthood in Relation to Number of Infection-Related Childhood Hospitalisations.

	% [N]		Model a		Model b	
	Cases	Controls	HR (95% CI)^1^	P-value	HR (95% CI)^2^	P-value
**Number of childhood hospitalisations with infection**						
**0**	55.0 [347]	70.9 [4474]	1	-	-	-
**1**	18.1 [114]	19.6 [1237]	1.0 (0.8–1.3)	0.9	1.0 (0.8–1.3)	0.9
**2**	8.7 [55]	4.9 [311]	1.7 (1.3–2.3)	<0.001	1.7 (1.3–2.3)	<0.001
**3+**	19.5 [115]	4.6 [288]	2.2 (1.7–2.9)	<0.001	2.2 (1.7–2.8)	<0.001

^1^ Model a: Hazard Ratios adjusted for year of birth, sex and Indigenous status

^2^ Model b: Hazard Ratios adjusted for year of birth, sex, Indigenous status and trauma-related childhood hospitalisation

A significant relationship between the number of IRH and CVD remained when individuals with only angina-related hospitalisation codes, with which there may be diagnostic uncertainty,[18] were removed. One hundred and thirty nine of 500 (28%) of non-angina CVD cases had two or more infection-related hospitalisations, compared with 599 (9.5%) controls; aHR, 1.97; 95% CI 1.6–2.5; *P* < 0.001. Analysis of specific clinical categories of CVD—ischaemic heart disease, peripheral vascular disease or ischaemic stroke, with acute admissions within each CVD group counted once—showed that the association with IRH remained. Two or more childhood IRH (634 of 6310 (10%) controls) were significantly associated with adult hospitalisation with an ischaemic heart disease-related diagnosis (109 of 345, 32%); aHR, 2.1; 95% CI 1.6–2.8; *P* < 0.001, an ischaemic stroke-related diagnosis (15 of 62, 24%) cases; aHR, 2.3; 95% CI 1.2–4.4; *P* < 0.05, and a peripheral vascular disease-related diagnosis (26 of 108, 14.4%) cases; aHR, 2.2; 95% CI 1.3–3.8; *P* < 0.01.

To assess the possible contribution of lower socioeconomic status in the entire cohort, we adjusted the analysis between childhood IRH and subsequent CVD for childhood trauma-related hospitalisations.[13,14] The relationship between the number of infection-related hospitalisations and adult CVD persisted; aHR for ≥3 IRH and CVD 2.2; 95% CI 1.7–2.8; *P* < 0.001 (Table 2 —Model b).

### Association between specific diagnostic groups of infection and adult CVD

We grouped the infectious disease codes into broad clinically-defined groups (S3 Table) to investigate whether particular types of infection (such as lower respiratory tract or gastrointestinal infections) had differential effects on subsequent CVD. 790 of the total 948 (83.3%) case IRH episodes and 2945 of 3299 (89.3%) control IRH could be grouped in this way. There was a significant dose-response association between the number of childhood group specific-IRH and adult CVD hospitalisation in all clinically-defined infection groups (Table 3). To assess the contribution of recurrent infections, we also analysed the relationship between childhood procedures relating to the consequences of otitis media and adult CVD. Childhood operations involving the tympanic membrane (cases N = 13; controls N = 53) were significantly associated with adult CVD in adjusted analyses (aHR1.79; 95% CI 1.0–3.2; *P* = 0.045).

**Table 3:** Adjusted Hazard Ratios^1^ for CVD Hospitalisation in Adulthood in Relation to Specific Clinical Category of Infection-Related Childhood Hospitalisations.

Infection subgroup	Cases% [n] (631)	Controls% [n] (6310)	HR^1^ ^,^ ^2^	95%CI	P-value
**Lower respiratory tract infection**					
0	90.0 [568]	94.9 [5990]			
1	7.0 [44]	4.1 [260]	1.17	0.9–1.6	0.33
2+	3.0 [19]	1.0 [60]	1.50	0.93–2.4	0.10
**Gastro-intestinal infection**					
0	81.8 [516]	90.5 [5710]			
1	10.3 [65]	7.5 [476]	0.92	0.7–1.2	0.57
2+	7.9 [50]	2.0 [124]	1.42	1.0–1.9	0.03
**Upper respiratory tract infection**					
0	78.3 [494]	88.3 [5574]			
1	14.7 [93]	9.2 [582]	1.42	1.1–1.8	0.002
2+	7.0 [44]	2.5 [154]	1.65	1.2–2.3	0.003
**Urinary tract infection**					
0	95.10 [600]	99.0 [6248]			
1	3.2 [20]	0.9 [55]	2.0	1.3–3.2	0.006
2+	1.7 [11]	0.1 [7]	4.2	2.3–7.7	<0.001
**Other viral infection**					
0	88.3 [579]	95.4 [6017]			
1	9.8 [45]	4.2 [266]	1.35	1.0–1.8	0.05
2+	1.9 [7]	0.4 [27]	1.55	0.7–3.3	0.25
**Skin and soft tissue infection**					
0	86.9 [548]	96.6 [6095]			
1	8.4 [53]	2.7 [172]	1.54	1.1–2.1	0.005
2+	4.8 [30]	0.7 [43]	2.09	1.4–3.1	<0.001

^1^ Hazard ratios are adjusted for year of birth, sex and Indigenous status.

^2^ Reference group is 0 hospitalisations, for each infection subgroup.

## Discussion

The role of infection in the development of atherosclerosis and as a risk factor for CVD has been debated for over 120 years.[19] The inflammatory processes central to the development of atherosclerosis begin in early life, although CVD is generally asymptomatic until at least early adulthood.[20] Infection occurs mainly in pre-school children and is the commonest reason for hospitalisation during childhood.[8] Most studies of the relationship between infection and CVD are in adults, using serological evidence of previous infection, data which lack sufficient granularity both on early life exposures, and on important confounding factors. This is the first study to examine the relationship between severe childhood infection and CVD in adulthood using longitudinal total population data in the same individuals. These demographic and health data are a unique resource and comprise over four decades of linked data on a large, stable total population with complete ascertainment of hospitalisations, robust data linkage, and repeated validation.[19,21] We have demonstrated a consistent and significant dose-response association between childhood IRH and CVD-related hospitalisations in early to mid-adulthood, which is independent of population-level risk factors. Given the effect sizes and the rigorous adjustment for confounding variables, we believe that the findings are robust. The data suggest that childhood infection, its treatment, and/or the host inflammatory response may lie on the causal pathway for atherosclerosis and CVD and may represent modifiable risk factors. Hospitalisation with infection may be a surrogate for those children who have a more marked inflammatory response to common childhood pathogens, or those that have a greater exposure to antibiotics in early life and childhood.

Where data were available, we adjusted for possible population-level risk factors that might confound the relationship between childhood IRH and adult CVD. Relative to the overall Western Australian population, there was an over-representation of Indigenous Australians among cases, reflecting the increased burden and earlier-onset of CVD in this population.[15] Indigenous children also have higher rates of IRH.[8] The significant association between childhood infection and CVD remained following adjustment for sex, year of birth, Indigenous status, and in analyses stratified on Indigenous status of cases and controls.

Low birth weight and lower socioeconomic status have been associated with increased risk of infection-related childhood hospitalisations,[20,22,23] and of CVD. [24] We adjusted for these parameters where data were available. The overall relationship between infection-related hospitalisation and CVD hospitalisation remained unaltered, although some associations became non-significant, reflecting the younger age of those in whom these data were available, which reduced the sample size; the maximum age of those with birth weight and socio-economic data was 29 years and CVD is uncommon before this age.

To assess possible effects of social deprivation on overall childhood hospitalisation in the entire sample, we also adjusted for trauma-related childhood hospitalisations. The association between childhood IRH and adult CVD remained unchanged following adjustment for childhood trauma admissions, indicating that confounding due to socioeconomic status is unlikely to account for the observed relationships.

The association between IRH and CVD risk was observed at a similar magnitude for all common clinical categories of infection, suggesting that childhood infection may have generic rather than pathogen-specific effects. As with IRH overall, a dose-response relationship was observed between the number of hospitalisations in childhood and adult CVD in all clinical categories of infection, and in most instances these associations were significant. This is in keeping with the concept that the overall infectious burden, rather than a specific pathogen, is a risk factor for CVD. Many pathogens are associated with CVD,[3] and over 50 bacterial species have been identified in atherosclerotic plaques.[25] Our findings suggest that a predominant pathogen is unlikely to be driving the association between childhood IRH and adult CVD. In addition the association with childhood IRH was observed for all CVD phenotypes, indicative of a non-specific association with atherosclerosis development.

Hospitalisation data do not allow investigation of chronic, milder infection characteristic of pathogens (e.g. *Chlamydophilia pneumoniae*, *Helicobacter pylori*, periodontal pathogens and CMV), which are suggested to contribute to atherosclerosis development and CVD. Investigation of these infections would require detailed prospective cohort studies. In an attempt to address milder, recurrent infections, we analysed surgical procedures that are a surrogate for recurrent childhood otitis media and observed a significant association with subsequent adult CVD.

Our study had unique strengths, particularly the use of longitudinal total population data over four decades and complete ascertainment of hospitalisation in a stable population. The use of hospitalisation as marker of infection and CVD severity is less influenced by health-seeking behaviour and social disadvantage than emergency department or primary care presentations,[26] and by practitioner-related variation in management.

The use of total population-linked data means there are some inherent limitations. Data linkage commenced in 1970 and as IRH were included from birth onwards (to capture the pre-school period when infection is most common and severe) cases were limited to those with earlier-onset CVD. Our cases therefore represent a relatively early-onset phenotype for CVD. Future studies will determine whether the relationship between infection-related hospitalisation and CVD alters as the population ages. The use of statutory data makes it impossible to assess individual-level CVD risk factors, such as hypercholesterolaemia, obesity, diabetes and smoking, which do not usually result in hospitalisation *per se*. These factors may interact with infection to increase the CVD risk and longitudinal studies are warranted.[21,27] Infection-related hospitalisations pre-dated rapid diagnostics and are not linked to microbiology data. Therefore diagnoses were ICD-coded and grouped clinically. Studies combining of diagnosis codes and laboratory data would identify pathogen-specific effects.[28] We could not assess the contribution of more common but less severe childhood infection to CVD risk. Infection-related hospitalisation may be a marker of a general propensity to mount a more marked inflammatory response to pathogens. Those children with IRH may also have a more marked inflammatory response during infections not necessitating hospitalisation. Repeated episodes of less marked infection-related inflammation may also contribute to atherosclerosis development. Indeed pro-atherosclerotic changes in oxidised-low density lipoprotein are observed during minor childhood infections, with subsequent increased carotid intima-media thickness and endothelial dysfunction,[29,30] although the long term CVD risk is unknown. Finally we were not able to investigate whether children with IRH had greater overall antibiotic exposures, as prescription data are not currently linked. Prospective studies across the life course that capture the full spectrum of severity of childhood infections, as well as infection-related treatment, and the relationship with adult CVD risk are warranted.

In summary our analysis of longitudinal total population over 40 years show that childhood IRH is significantly associated with adult CVD hospitalisation in a dose-response manner. The effect is observed with all common infections and manifestations of CVD. Reduction of the childhood infection burden, especially in those with other CVD risk factors, may reduce the burden of CVD in adulthood.

## Clinical Perspectives

- Childhood infection results in repeated inflammatory responses. The contribution of childhood infection to the development of atherosclerosis, a chronic inflammatory condition that begins in early life, and to clinical CVD risk is unknown.
- Using longitudinal population-derived data on the same individuals over four decades, we show a significant independent dose-response relationship between the number of hospital admissions with infection in childhood, and an increased risk of CVD in adulthood. This relationship was observed for all clinical types of infection, and with ischaemic heart disease, ischaemic stroke and peripheral vascular disease
- Childhood infection, its treatment, and/or the resultant inflammatory response, may be under-appreciated risk factors for adult CVD. Reducing the frequency of severe infection in childhood, and addressing traditional cardiovascular risk factors in those with a significant history of infection may decrease the burden of CVD in adulthood.

## Supporting Information

S1 Table ICD9 and ICD10 diagnosis codes for cardiovascular disease groups. (DOC)

S2 Table ICD9 and ICD10 diagnosis codes included for each infection subgroup and for ‘any infection’ group. (DOCX)

S3 Table Adjusted hazard ratios for cardiovascular disease hospitalisation in adulthood in relation to number of infection-related childhood hospitalisations on subjects with birth weight data. (DOCX)

S4 Table ICD9 and ICD10 procedure codes for chronic or recurrent middle ear infection. (DOCX)
