# Supplementary material for: Childhood Hospitalisation with Infection and Cardiovascular Disease in Early-Mid Adulthood: A Longitudinal Population-Based Study
Source: PLoS One. 2015 May 4;10(5):e0125342. doi: 10.1371/journal.pone.0125342 (PMC4418819; doi:10.1371/journal.pone.0125342)
Supplement: S1 Table — (DOC) [file pone.0125342.s001.doc]

**TABLE S1**

Table S1: ICD9 and ICD10 Diagnosis Codes for cardiovascular Disease Groups

| **CVD subset** | **ICD9** | **ICD10** |
| --- | --- | --- |
| Stroke | 433,433.01,433.1,433.11,433.2,433.21,433.3,433.31,433.8,433.81,433.9,433.91,434,434.01,434.1,434.11,434.9,434.91,436,437 | I63.0,I63.2,I64,I65.0,I65.1,I65.2,I65.3,I65.8,I65.9,I67.2 |
| Ischemic Heart Disease | 410,410.01,410.02,410.1,410.11,410.12,410.2,410.21,410.22,410.3,410.31,410.32,410.4,410.41,410.42,410.5,410.51,410.52,410.6,410.61,410.62,410.7,410.71,410.72,410.8,410.81,410.82,410.9,410.91,410.92,411,411.1,411.8,411.81,411.89,412,414,414.01,414.02,414.03,414.04,414.05,414.06,414.07,414.11,414.12,414.19,414.2,414.8,414.9 | I20.0,I21.0,I21.1,I21.2,I21.3,I21.4,I21.9,I22.0,I22.1,I22.8,I22.9,I23.0,I23.1,I23.2,I23.3,I23.4,I23.5,I23.6,I24.0,I24.1,I24.8,I24.9,I25.0,I25.10,I25.11,I25.12,I25.13,I25.2,I25.3,I25.4,I25.5,I25.6,I25.8,I25.9,429.2,429.79,I23.8 |
| Peripheral Vascular Disease | 440,440.1,440.2,440.21,440.22,440.23,440.24,440.29,440.3,440.31,440.32,440.4,440.8,440.9,441,441.01,441.02,441.03,441.1,441.2,441.3,441.4,441.5,441.6,441.7,441.9,442,442.1,442.2,442.3,442.81,442.82,442.83,442.84,442.89,442.9,443.1,443.21,443.22,443.23,443.24,443.29,443.89,443.9 | I70.0,I70.1,I70.2,I70.20,I70.21,I70.22,I70.23,I70.24,I70.8,I70.9,I71.0,I71.00,I71.01,I71.02,I71.03,I71.1,I71.2,I71.3,I71.4,I71.5,I71.6,I71.8,I71.9,I72.0,I72.1,I72.2,I72.3,I72.4,I72.8,I72.9,I73.1,I73.8,I73.9 |
| Angina | 413.1,413.9 | I20,I20.0,I20.1,I20.8,I20.9 |
