# Supplementary material for: Childhood Hospitalisation with Infection and Cardiovascular Disease in Early-Mid Adulthood: A Longitudinal Population-Based Study
Source: PLoS One. 2015 May 4;10(5):e0125342. doi: 10.1371/journal.pone.0125342 (PMC4418819; doi:10.1371/journal.pone.0125342)
Supplement: S2 Table — (DOCX) [file pone.0125342.s002.docx]

**TABLE S2:** ICD9 and ICD10 Diagnosis Codes included for each infection subgroup and for “any infection” group

| **Abbreviation** | **Infectious Disease** | **ICD9** | **ICD10** |
| --- | --- | --- | --- |
| LRTI | Acute lower respiratory tract infection | 003.22,006.4,010.00 ,010.01 ,010.02 ,010.03 ,010.04 ,010.05 ,010.06 ,010.10 ,010.11 ,010.12 ,010.13 ,010.14 ,010.15 ,010.16 ,010.80 ,010.81 ,010.82 ,010.83 ,010.84 ,010.85 ,010.86 ,010.90 ,010.91 ,010.92 ,010.93 ,010.94 ,010.95 ,010.96 ,011.00 ,011.01 ,011.02 ,011.03 ,011.04 ,011.05 ,011.06 ,011.10 ,011.11 ,011.12 ,011.13 ,011.14 ,011.15 ,011.16 ,011.20 ,011.21 ,011.22 ,011.23 ,011.24 ,011.25 ,011.26 ,011.30 ,011.31 ,011.32 ,011.33 ,011.34 ,011.35 ,011.36 ,011.40 ,011.41 ,011.42 ,011.43 ,011.44 ,011.45 ,011.46 ,011.50 ,011.51 ,011.52 ,011.53 ,011.54 ,011.55 ,011.56 ,011.60 ,011.61 ,011.62 ,011.63 ,011.64 ,011.65 ,011.66 ,011.70 ,011.71 ,011.72 ,011.73 ,011.74 ,011.75 ,011.76 ,011.80 ,011.81 ,011.82 ,011.83 ,011.84 ,011.85 ,011.86 ,011.90 ,011.91 ,011.92 ,011.93 ,011.94 ,011.95 ,011.96 ,012.00 ,012.01 ,012.02 ,012.03 ,012.04 ,012.05 ,012.06 ,012.10 ,012.11 ,012.12 ,012.13 ,012.14 ,012.15 ,012.16 ,018.00 ,018.01 ,018.02 ,018.03 ,018.04 ,018.05 ,018.06 ,018.80 ,018.81 ,018.82 ,018.83 ,018.84 ,018.85 ,018.86 ,018.90 ,018.91 ,018.92 ,018.93 ,018.94 ,018.95 ,018.96 ,020.3,020.4,020.5,021.2,022.1,024,031.0,033.0,033.1,033.8,033.9,039.1,052.1,055.1,073.0,073.7,073.8,073.9,079.0,079.6,079.82,112.4,114.0,114.4,114.5,115.05,115.10,115.15,115.90,115.95,130.4,136.3,464.4,466.0,466.1,466.11,466.19,480.0,480.1,480.2,480.3,480.8,480.9,481,481.9,482.0,482.1,482.2,482.3,482.30,482.31,482.32,482.39,482.4,482.40,482.41,482.42,482.49,482.8,482.81,482.82,482.83,482.84,482.89,482.9,483,483.0,483.1,483.8,484.1,484.3,484.5,484.6,484.7,484.8,485,486,487.0,487.1,487.8,488.01,488.02,488.11,488.12,488.81,488.82,490,491.0,491.1,491.2,491.20,491.21,491.22,491.8,491.9,494,494.0,494.1,510.0,510.9,513.0,513.1,517.1,770.0 | A06.5+,A15.0,A15.1,A15.2,A15.3,A15.4,A15.5,A15.6,A15.7,A15.8,A15.9,A16.0,A16.1,A16.2,A16.3,A16.4,A16.5,A16.7,A16.8,A16.9,A19.2,A19.8,A19.9,A20.2,A21.2,A22.1,A24.0,A31.0,A37.0,A37.1,A37.8,A37.9,A42.0,A48.1,A70,A70+,B01.2+,B05.2+,B34.0,B37.1,B38.0,B38.1,B38.2,B39.0,B39.1,B39.2,B39.3,B39.4,B39.5,B39.9,B40.0,B40.1,B40.2,B40.3,B40.7,B40.8,B41.0,B42.0+,B44.0,B44.1,B45.0,B58.3+,B59,J05.0,J09,J10.0,J10.1,J11.0,J11.1,J12.0,J12.1,J12.2,J12.3,J12.8,J12.9,J13,J14,J15.0,J15.1,J15.2,J15.3,J15.4,J15.5,J15.6,J15.7,J15.8,J15.9,J16.0,J16.8,J17.0*,J17.1*,J17.2*,J17.3,J17.8*,J18.0,J18.1,J18.8,J18.9,J20.0,J20.1,J20.2,J20.3,J20.4,J20.5,J20.6,J20.7,J20.8,J20.9,J21.0,J21.1,J21.8,J21.9,J22,J22.0,J40,J41.0,J41.1,J41.8,J42,J44.0,J47,J65,J85.0,J85.1,J85.2,J86.0,J86.9,P23.0,P23.2,P23.3,P23.4,P23.5,P23.6,P23.8,P23.9 |
| GI | Gastrointestinal infections | 001.0,001.1,001.9,002.0,002.1,002.2,002.3,002.9,003.0,003.20,003.29,003.8,003.9,004.0,004.1,004.2,004.3,004.8,004.9,005.0,005.1,005.2,005.3,005.4,005.8,005.81,005.89,005.9,006.0,006.1,006.2,006.3,006.8,006.9,007.0,007.1,007.2,007.3,007.4,007.5,007.8,007.9,008.0,008.00,008.01,008.02,008.03,008.04,008.09,008.1,008.2,008.3,008.41,008.42,008.43,008.44,008.45,008.46,008.47,008.49,008.5,008.6,008.61,008.62,008.63,008.64,008.65,008.66,008.67,008.69,008.8,009.0,009.1,009.2,009.3,021.1,022.2,032.83,039.2,040.2,041.86,078.82,112.85,112.89,123.6,123.9,126.0,126.9,127.1,127.2,558.9 | A00.0,A00.1,A00.9,A01.0,A01.1,A01.2,A01.3,A01.4,A02.0,A02.2+,A02.8,A02.9,A03.0,A03.1,A03.2,A03.3,A03.8,A03.9,A04.0,A04.1,A04.2,A04.3,A04.4,A04.5,A04.6,A04.7,A04.8,A04.9,A05.0,A05.1,A05.2,A05.3,A05.4,A05.8,A05.9,A06.0,A06.1,A06.2,A06.3,A06.4,A06.9,A07.0,A07.1,A07.2,A07.3,A07.8,A07.9,A08.0,A08.1,A08.2,A08.3,A08.4,A08.5,A09,A09.0,A09.9,A21.3,A22.2,B37.88,B71.0,B71.9,B76.0,B78.9,B81.0,B96.81,K52.8 |
| URTI | Upper respiratory tract infection (including Otitis Media) | 032.0,032.1,032.2,032.3,034.0,040.1,055.2,079.3,381.00,381.01,381.02,381.03,381.10,381.19,381.20,381.29,381.3,381.4,382.00,382.01,382.02,382.1,382.2,382.3,382.4,382.9,383.00,383.01,383.02,383.1,383.20,383.21,383.22,383.30,383.31,383.32,383.33,383.89,383.9,384.00,384.01,384.09,384.20,384.21,384.22,384.23,384.24,384.25,386.3,386.30,386.31,386.32,386.33,386.34,386.35,388.6,460,461.0,461.1,461.2,461.3,461.8,461.9,462,463,464.0,464.00,464.01,464.10,464.11,464.20,464.21,464.30,464.31,464.50,464.51,465.0,465.8,465.9,473.0,473.1,473.2,473.3,473.8,473.9,474.0,474.00,474.01,474.02,475,476.0,476.1,478.21,478.22,478.24,478.29,478.71 | A36.0,A36.1,A36.2,B05.3+,H65.0,H65.1,H65.2,H65.3,H65.4,H65.9,H66.0,H66.1,H66.2,H66.3,H66.4,H66.9,H67.0,H67.8*,H68.0,H70.0,H70.1,H70.2,H70.8,H70.9,H72.0,H72.1,H72.2,H72.8,H72.9,H73.0,H75.0,H83.0,H92.1,J00,J01.0,J01.1,J01.2,J01.3,J01.4,J01.8,J01.9,J02.0,J02.8,J02.9,J03.0,J03.8,J03.9,J04.0,J04.1,J04.2,J05.1,J06.0,J06.8,J06.9,J32.0,J32.1,J32.2,J32.3,J32.4,J32.8,J32.9,J34.0,J35.0,J36 |
| UTI | Urinary tract infection (including STI) | 032.84,091.0,091.1,091.2,098.0,098.10,098.11,098.12,098.13,098.14,098.15,098.16,098.17,098.19,098.2,098.30,098.31,098.32,098.33,098.34,098.35,098.36,098.37,098.39,099.0,099.1,099.2,099.41,099.49,099.50,099.51,099.52,099.53,099.54,099.55,099.56,099.59,112.1,112.2,590.2,590.81,590.9,595.0,595.89,597.0,598.00,599.0,601.0,601.2,603.1,604.0,614.0,614.1,614.2,614.3,614.4,614.5,614.6,614.7,614.8,614.9,615.0,615.1,615.9,616.0,616.10,616.11,616.2,616.3,616.4,634.00,634.01,634.02,646.60,646.61,646.62,646.63,646.64,647.00,647.01 ,647.02 ,647.03 ,647.04 ,647.10 ,647.11 ,647.12 ,647.13 ,647.14 ,647.20 ,647.21,647.22 ,647.23 ,647.24 ,771.82 | A36.8+,A51.0,A51.1,A51.2,A51.3,A51.3+,A51.4,A51.5,A51.9,A52.9,A53.0,A53.9,A54.0,A54.1,A54.2+,A54.3,A54.3+,A54.4+,A54.5,A54.6,A54.8,A54.8+,A54.9,A55,A56.0,A56.1,A56.2,A56.3,A56.4,A56.8,A57,A58,A59.0,A59.0+,A60.0,A60.1,A60.9,A63.0,A63.8,A64,B37.3+,B37.4+,N13.6,N15.1,N30.0,N30.8,N34.0,N35.1,N39.0,N41.0,N41.2,N43.1,N45.0,N45.9,N51.2,N70.0,N73.0,N73.1,N73.2,N73.3,N73.4,N73.9,N74.2,N74.3,N74.4,N75.0,N75.1,N76.0,N76.4,O03.0,O03.5,O08.0,O26.4,O86.2,O98.1,O98.2,P39.3 |
| OTHER VIRAL | Viral infection not otherwise specified | 042,042.0,042.1,042.2,042.9,043.0,043.1,043.2,043.3,043.9,044.0,044.9,045.00,045.01,045.02,045.03,045.10,045.11,045.12,045.13,045.20,045.21,045.22,045.23,045.90,045.91,045.92,045.93,046.8,046.9,047.0,047.1,047.8,047.9,048,049.0,049.1,049.8,049.9,050.0,050.1,050.2,050.9,051.0,051.01,051.02,051.1,051.2,051.9,052.0,052.2,052.7,052.8,052.9,053.0,053.10,053.11,053.12,053.13,053.14,053.19,053.20,053.21,053.22,053.29,053.71,053.79,053.8,053.9,054.10,054.11,054.12,054.13,054.19,054.2,054.3,054.40,054.41,054.42,054.43,054.44,054.49,054.5,054.6,054.71,054.72,054.73,054.74,054.79,054.8,054.9,055.0,055.71,055.79,055.8,055.9,056.00,056.01,056.09,056.71,056.79,056.8,056.9,057.0,057.8,057.9,058.10,058.11,058.12,058.21,058.29,058.81,058.82,058.89,059.00,059.01,059.09,059.10,059.11,059.12,059.19,059.20,059.21,059.22,059.8,059.9,060.0,060.1,060.9,061,062.0,062.1,062.2,062.3,062.4,062.5,062.8,062.9,063.0,063.1,063.2,063.8,063.9,064,065.0,065.1,065.2,065.3,065.4,065.8,065.9,066.0,066.1,066.2,066.3,066.4,066.40,066.41,066.42,066.49,066.8,066.9,070.0,070.1,070.2,070.20,070.21,070.22,070.23,070.3,070.30,070.31,070.32,070.33,070.4,070.41,070.42,070.43,070.44,070.49,070.5,070.51,070.52,070.53,070.54,070.59,070.6,070.70,070.71,070.9,071,072.0,072.1,072.2,072.3,072.71,072.72,072.79,072.8,072.9,074.0,074.1,074.20,074.21,074.22,074.23,074.3,074.8,075,077.1,077.2,077.3,077.4,077.8,077.99,078.0,078.1,078.10,078.11,078.12,078.19,078.2,078.4,078.5,078.6,078.7,078.81,078.89,079.1,079.2,079.4,079.50,079.51,079.52,079.53,079.59,079.81,079.83,079.89,079.99,323.0,323.02,488.09,488.19,488.89,573.1,647.50,647.51,647.52,647.53,647.54,647.60,647.61,647.62,647.63,647.64,695.3,711.50,711.51,711.52,711.53,711.54,711.55,711.56,711.57,711.58,711.59,771.0,771.1,790.8,795.8 | A80.1,A80.2,A80.3,A80.4,A80.9,A81.8,A81.9,A82.0,A82.1,A82.9,A83.0,A83.1,A83.2,A83.3,A83.4,A83.5,A83.6,A83.8,A83.9,A84.0,A84.1,A84.8,A84.9,A85.0,A85.1,A85.2,A85.8,A86,A87.0+,A87.1+,A87.2,A87.8,A87.9,A88.0,A88.8,A89,A90,A91,A92.0,A92.1,A92.2,A92.3,A92.4,A92.8,A92.9,A93.0,A93.2,A93.8,A94,A95.0,A95.1,A95.9,A96.0,A96.1,A96.2,A96.8,A96.9,A98.0,A98.1,A98.2,A98.3,A98.4,A98.5,A98.8,A99,B00.1,B00.2,B00.3+,B00.4+,B00.5+,B00.7,B00.8,B00.9,B01.0,B01.1+,B01.8,B01.9,B02.0,B02.1+,B02.2+,B02.3+,B02.7,B02.8,B02.9,B03,B04,B05.0+,B05.1,B05.4,B05.8,B05.9,B06.0+,B06.8,B06.9,B07,B08.0,B08.1,B08.2,B08.3,B08.4,B08.5,B08.8,B09,B15.0,B15.9,B16.0,B16.1,B16.2,B16.9,B17.0,B17.1,B17.2,B17.8,B17.9,B18.0,B18.1,B18.2,B18.8,B18.9,B19.0,B19.9,B20.0,B20.1,B20.2,B20.3,B20.4,B20.5,B20.6,B20.7,B20.8,B20.9,B21.0,B21.1,B21.2,B21.3,B21.7,B21.8,B21.9,B22.0,B22.1,B22.2,B22.7,B23.0,B23.1,B23.2,B23.8,B24,B25.0,B25.1,B25.2,B25.8,B25.9,B26.0+,B26.1+,B26.2+,B26.3+,B26.8,B26.8+,B26.9,B27.0,B27.1,B27.8,B27.9,B30.0+,B30.1+,B30.2+,B30.3+,B30.8+,B30.9,B33.0,B33.1,B33.2,B33.3,B33.4,B33.8,B34.1,B34.2,B34.3,B34.4,B34.8,B34.9,B97.0,B97.1,B97.2,B97.3,B97.4,B97.5,B97.6,B97.7,B97.8,G02.0*,G05.1*,H19.1,I41.1,J10.8,J11,J11.8,M01.4,M01.50*,M01.51*,M01.52*,M01.53*,M01.54*,M01.55*,M01.56*,M01.57*,M01.58*,M01.59*,O98.4,O98.5,P35.0,P35.1,P35.2,P35.3,P35.8,P35.9,V08,Z21 |
| SKIN, BACT, SOFT TISSUE | Skin and soft tissue infections | 006.6,020.1,022.0,031.1,032.85,035,039.0,039.4,040.0,040.42,040.81,054.0,110.0,110.1,110.2,110.3,110.4,110.5,110.6,110.8,110.9,111.0,111.1,111.2,111.3,111.8,111.9,112.3,132.0,132.1,132.2,132.3,132.9,133.0,133.8,133.9,134.0,134.1,134.2,134.8,134.9,376.01,380.10,380.11,675.00,675.01,675.02 ,675.03,675.04,675.10,675.11,675.12,675.13,675.14,675.20,675.21,675.22,675.23,675.24,675.80,675.81,675.82,675.83,675.84,675.90,675.91,675.92,675.93,675.94,680.0,680.1,680.2,680.3,680.4,680.5,680.6,680.7,680.8,680.9,681.00,681.01,681.02,681.10,681.11,681.9,682.0,682.1,682.2,682.3,682.4,682.5,682.6,682.7,682.8,682.9,684,685.0,685.1,686.0,686.00,686.01,686.09,686.1,686.8,686.9,690.8,694.0,694.1,694.2,694.3,771.5 | A06.7,A31.1,A46,B00.0,B35.0,B35.1,B35.2,B35.3,B35.4,B35.5,B35.6,B35.8,B35.9,B36.0,B36.1,B36.2,B36.3,B36.8,B37.2,B85.0,B85.1,B85.2,B85.3,B85.4,B86,B87.9,B88.0,B88.1,B88.2,B88.3,B88.8,B88.9,H60.3,L00,L01.0,L02.0,L02.1,L02.2,L02.3,L02.4,L02.8,L02.9,L03.01,L03.02,L03.10,L03.11,L03.2,L03.3,L03.8,L03.9,L05.0,L05.9,L08.0,L08.1,L08.8,L08.9,L13.0,L30.3,L88,M60.09,M63.0,M63.1,M63.2,M65.0,M65.1,M68.0,M71.0,M71.1,O91.00,O91.10,P39.0,P39.4 |
| ANY Infection | Any infection | 001.0,001.1,001.9,002.0,002.1,002.2,002.3,002.9,003.0,003.1,003.20,003.21,003.22,003.23,003.24,003.29,003.8,003.9,004.0,004.1,004.2,004.3,004.8,004.9,005.0,005.1,005.2,005.3,005.4,005.8,005.81,005.89,005.9,006.0,006.1,006.2,006.3,006.4,006.5,006.6,006.8,006.9,007.0,007.1,007.2,007.3,007.4,007.5,007.8,007.9,008.0,008.00,008.01,008.02,008.03,008.04,008.09,008.1,008.2,008.3,008.41,008.42,008.43,008.44,008.45,008.46,008.47,008.49,008.5,008.6,008.61,008.62,008.63,008.64,008.65,008.66,008.67,008.69,008.8,009.0,009.1,009.2,009.3,010.00,010.01,010.02,010.03,010.04,010.05,010.06,010.10,010.11,010.12,010.13,010.14,010.15,010.16,010.80,010.81,010.82,010.83,010.84,010.85,010.86,010.90,010.91,010.92,010.93,010.94,010.95,010.96,011.00,011.01,011.02,011.03,011.04,011.05,011.06,011.10,011.11,011.12,011.13,011.14,011.15,011.16,011.20,011.21,011.22,011.23,011.24,011.25,011.26,011.30,011.31,011.32,011.33,011.34,011.35,011.36,011.40,011.41,011.42,011.43,011.44,011.45,011.46,011.50,011.51,011.52,011.53,011.54,011.55,011.56,011.60,011.61,011.62,011.63,011.64,011.65,011.66,011.70,011.71,011.72,011.73,011.74,011.75,011.76,011.80,011.81,011.82,011.83,011.84,011.85,011.86,011.90,011.91,011.92,011.93,011.94,011.95,011.96,012.00,012.01,012.02,012.03,012.04,012.05,012.06,012.10,012.11,012.12,012.13,012.14,012.15,012.16,012.20,012.21,012.22,012.23,012.24,012.25,012.26,012.30,012.31,012.32,012.33,012.34,012.35,012.36,012.80,012.81,012.82,012.83,012.84,012.85,012.86,013.00,013.01,013.02,013.03,013.04,013.05,013.06,013.10,013.11,013.12,013.13,013.14,013.15,013.16,013.20,013.21,013.22,013.23,013.24,013.25,013.26,013.30,013.31,013.32,013.33,013.34,013.35,013.36,013.40,013.41,013.42,013.43,013.44,013.45,013.46,013.50,013.51,013.52,013.53,013.54,013.55,013.56,013.60,013.61,013.62,013.63,013.64,013.65,013.66,013.80,013.81,013.82,013.83,013.84,013.85,013.86,013.90,013.91,013.92,013.93,013.94,013.95,013.96,014.00,014.01,014.02,014.03,014.04,014.05,014.06,014.80,014.81,014.82,014.83,014.84,014.85,014.86,015.00,015.01,015.02,015.03,015.04,015.05,015.06,015.10,015.11,015.12,015.13,015.14,015.15,015.16,015.20,015.21,015.22,015.23,015.24,015.25,015.26,015.50,015.51,015.52,015.53,015.54,015.55,015.56,015.60,015.61,015.62,015.63,015.64,015.65,015.66,015.70,015.71,015.72,015.73,015.74,015.75,015.76,015.80,015.81,015.82,015.83,015.84,015.85,015.86,015.90,015.91,015.92,015.93,015.94,015.95,015.96,016.00,016.01,016.02,016.03,016.04,016.05,016.06,016.10,016.11,016.12,016.13,016.14,016.15,016.16,016.20,016.21,016.22,016.23,016.24,016.25,016.26,016.30,016.31,016.32,016.33,016.34,016.35,016.36,016.40,016.41,016.42,016.43,016.44,016.45,016.46,016.50,016.51,016.52,016.53,016.54,016.55,016.56,016.60,016.61,016.62,016.63,016.64,016.65,016.66,016.70,016.71,016.72,016.73,016.74,016.75,016.76,016.90,016.91,016.92,016.93,016.94,016.95,016.96,017.00,017.01,017.02,017.03,017.04,017.05,017.06,017.10,017.11,017.12,017.13,017.14,017.15,017.16,017.20,017.21,017.22,017.23,017.24,017.25,017.26,017.30,017.31,017.32,017.33,017.34,017.35,017.36,017.40,017.41,017.42,017.43,017.44,017.45,017.46,017.50,017.51,017.52,017.53,017.54,017.55,017.56,017.60,017.61,017.62,017.63,017.64,017.65,017.66,017.70,017.71,017.72,017.73,017.74,017.75,017.76,017.80,017.81,017.82,017.83,017.84,017.85,017.86,017.90,017.91,017.92,017.93,017.94,017.95,017.96,018.00,018.01,018.02,018.03,018.04,018.05,018.06,018.80,018.81,018.82,018.83,018.84,018.85,018.86,018.90,018.91,018.92,018.93,018.94,018.95,018.96,020.0,020.1,020.2,020.3,020.4,020.5,020.8,020.9,021.0,021.1,021.2,021.3,021.8,021.9,022.0,022.1,022.2,022.3,022.8,022.9,023.0,023.1,023.2,023.3,023.8,023.9,024,025,026.0,026.1,026.9,027.0,027.1,027.2,027.8,027.9,030.0,030.1,030.2,030.3,030.8,030.9,031.0,031.1,031.2,031.8,031.9,032.0,032.1,032.2,032.3,032.81,032.83,032.84,032.85,032.89,032.9,033.0,033.1,033.8,033.9,034.0,034.1,035,036.0,036.1,036.2,036.3,036.40 ,036.41,036.42,036.43,036.81,036.82,036.89,036.9,037,038.0,038.1,038.10,038.11,038.12,038.19,038.2,038.3,038.40,038.41,038.42,038.43,038.44,038.49,038.8,038.9,039.0,039.1,039.2,039.3,039.4,039.8,039.9,040.0,040.1,040.2,040.3,040.41,040.42,040.81,040.82,040.89,041.0,041.00,041.01,041.02,041.03,041.04,041.05,041.09,041.1,041.10,041.11,041.12,041.19,041.2,041.3,041.4,041.5,041.6,041.7,041.8,041.81,041.82,041.83,041.84,041.85,041.86,041.89,041.9,042,042.0,042.1,042.2,042.9,043.0,043.1,043.2,043.3,043.9,044.0,044.9,045.00,045.01,045.02,045.03,045.10,045.11,045.12,045.13,045.20,045.21,045.22,045.23,045.90,045.91,045.92,045.93,046.0,046.1,046.11,046.19,046.3,046.71,046.72,046.79,046.8,046.9,047.0,047.1,047.8,047.9,048,049.0,049.1,049.8,049.9,050.0,050.1,050.2,050.9,051.0,051.01,051.02,051.1,051.2,051.9,052.0,052.1,052.2,052.7,052.8,052.9,053.0,053.10,053.11,053.12,053.13,053.14,053.19,053.20,053.21,053.22,053.29,053.71,053.79,053.8,053.9,054.0,054.10,054.11,054.12,054.13,054.19,054.2,054.3,054.40,054.41,054.42,054.43,054.44,054.49,054.5,054.6,054.71,054.72,054.73,054.74,054.79,054.8,054.9,055.0,055.1,055.2,055.71,055.79,055.8,055.9,056.00,056.01,056.09,056.71,056.79,056.8,056.9,057.0,057.8,057.9,058.10,058.11,058.12,058.21,058.29,058.81,058.82,058.89,059.00,059.01,059.09,059.10,059.11,059.12,059.19,059.20,059.21,059.22,059.8,059.9,060.0,060.1,060.9,061,062.0,062.1,062.2,062.3,062.4,062.5,062.8,062.9,063.0,063.1,063.2,063.8,063.9,064,065.0,065.1,065.2,065.3,065.4,065.8,065.9,066.0,066.1,066.2,066.3,066.4,066.40,066.41,066.42,066.49,066.8,066.9,070.0,070.1,070.2,070.20,070.21,070.22,070.23,070.3,070.30,070.31,070.32,070.33,070.4,070.41,070.42,070.43,070.44,070.49,070.5,070.51,070.52,070.53,070.54,070.59,070.6,070.70,070.71,070.9,071,072.0,072.1,072.2,072.3,072.71,072.72,072.79,072.8,072.9,073.0,073.7,073.8,073.9,074.0,074.1,074.20,074.21,074.22,074.23,074.3,074.8,075,076.0,076.1,076.9,077.0,077.1,077.2,077.3,077.4,077.8,077.9,077.98,077.99,078.0,078.1,078.10,078.11,078.12,078.19,078.2,078.3,078.4,078.5,078.6,078.7,078.81,078.82,078.88,078.89,079.0,079.1,079.2,079.3,079.4,079.50,079.51,079.52,079.53,079.59,079.6,079.8,079.81,079.82,079.83,079.88,079.89,079.9,079.98,079.99,080,081.0,081.1,081.2,081.9,082.0,082.1,082.2,082.3,082.40,082.41,082.49,082.8,082.9,083.0,083.1,083.2,083.8,083.9,084.0,084.1,084.2,084.3,084.4,084.5,084.6,084.7,084.8,084.9,085.0,085.1,085.2,085.3,085.4,085.5,085.9,086.0,086.1,086.2,086.3,086.4,086.5,086.9,087.0,087.1,087.9,088.0,088.8,088.81,088.82,088.89,088.9,090.0,090.1,090.2,090.3,090.40,090.41,090.42,090.49,090.5,090.6,090.7,090.9,091.0,091.1,091.2,091.3,091.4,091.50,091.51,091.52,091.61,091.62,091.69,091.7,091.81,091.82,091.89,091.9,092.0,092.9,093.0,093.1,093.20,093.21,093.22,093.23,093.24,093.81,093.82,093.89,093.9,094.0,094.1,094.2,094.3,094.81,094.82,094.83,094.84,094.85,094.86,094.87,094.89,094.9,095.0,095.1,095.2,095.3,095.4,095.5,095.6,095.7,095.8,095.9,096,097.0,097.1,097.9,098.0,098.10,098.11,098.12,098.13,098.14,098.15,098.16,098.17,098.19,098.2,098.30,098.31,098.32,098.33,098.34,098.35,098.36,098.37,098.39,098.40,098.41,098.42,098.43,098.49,098.50,098.51,098.52,098.53,098.59,098.6,098.7,098.81,098.82,098.83,098.84,098.85,098.86,098.89,099.0,099.1,099.2,099.4,099.40,099.41,099.49,099.50,099.51,099.52,099.53,099.54,099.55,099.56,099.59,099.8,099.9,100.0,100.81,100.89,100.9,101,102.0,102.1,102.2,102.3,102.4,102.5,102.6,102.7,102.8,102.9,103.0,103.1,103.2,103.3,103.9,104.0,104.8,104.9,110.0,110.1,110.2,110.3,110.4,110.5,110.6,110.8,110.9,111.0,111.1,111.2,111.3,111.8,111.9,112.0,112.1,112.2,112.3,112.4,112.5,112.81,112.82,112.83,112.84,112.85,112.89,112.9,114.0,114.1,114.2,114.3,114.4,114.5,114.9,115.00,115.01,115.02,115.05,115.09,115.10,115.11,115.12,115.15,115.19,115.90,115.91,115.92,115.95,115.99,116.0,116.1,116.2,117.0,117.1,117.2,117.3,117.4,117.5,117.6,117.7,117.8,117.9,118,120.0,120.1,120.2,120.3,120.8,120.9,121.0,121.1,121.2,121.3,121.4,121.5,121.6,121.8,121.9,122.0,122.1,122.2,122.3,122.4,122.5,122.6,122.7,122.8,122.9,123.0,123.1,123.2,123.3,123.4,123.5,123.6,123.8,123.9,124,125.0,125.1,125.2,125.3,125.4,125.5,125.6,125.7,125.9,126.0,126.1,126.2,126.3,126.8,126.9,127.0,127.1,127.2,127.3,127.4,127.5,127.6,127.7,127.8,127.9,128.0,128.1,128.8,128.9,129,130.0,130.1,130.2,130.4,130.5,130.7,130.8,130.9,131.00,131.01,131.02,131.03,131.09,131.8,131.9,132.0,132.1,132.2,132.3,132.9,133.0,133.8,133.9,134.0,134.1,134.2,134.8,134.9,135,136.0,136.2,136.21,136.29,136.3,136.4,136.5,136.8,136.9,283.11 ,320.0,320.1,320.2,320.3,320.7,320.8,320.81,320.82,320.89,320.9,321.0,321.1,321.2,321.3,321.4,321.8,322.0,322.1,322.2,322.9,323.0,323.01,323.02,323.1,323.2,323.4,323.41,323.42,323.6,323.61,323.62,323.63,323.8,323.81,323.82,323.9,324.0,324.1,324.9,325,326,357.0,360.00,360.01,360.02,360.03,360.04,360.11,360.12,360.13,360.14,360.19,363.20,364.03,364.05,370.55,372.00,372.01,372.02,372.03,372.04,372.15,372.20,373.00,373.01,373.02,373.11,373.12,373.13,373.2,373.4,373.5,373.6,376.01,376.02,376.03,376.04,376.13,379.60,379.61,379.62,379.63,380.10,380.11,380.12,380.13,380.14,380.15,380.16,380.23,381.00,381.01,381.02,381.03,381.10,381.19,381.20,381.29,381.3,381.4,382.00,382.01,382.02,382.1,382.2,382.3,382.4,382.9,383.00,383.01,383.02,383.1,383.20,383.21,383.22,383.30,383.31,383.32,383.33,383.89,383.9,384.00,384.01,384.09,384.20,384.21,384.22,384.23,384.24,384.25,386.3,386.30 ,386.31 ,386.32 ,386.33 ,386.34 ,386.35 ,388.6,390,391.0,391.1,391.2,391.8,391.9,392.0,392.9,393,421.0,449,460,461.0,461.1,461.2,461.3,461.8,461.9,462,463,464.0,464.00,464.01,464.10,464.11,464.20,464.21,464.30,464.31,464.4,464.50,464.51,465.0,465.8,465.9,466.0,466.1,466.11,466.19,473.0,473.1,473.2,473.3,473.8,473.9,474.0,474.00,474.01,474.02,475,476.0,476.1,478.21,478.22,478.24,478.29,478.71,480.0,480.1,480.2,480.3,480.8,480.9,481,481.9,482.0,482.1,482.2,482.3,482.30,482.31,482.32,482.39,482.4,482.40,482.41,482.42,482.49,482.8,482.81,482.82,482.83,482.84,482.89,482.9,483,483.0,483.1,483.8,484.1,484.3,484.5,484.6,484.7,484.8,485,486,487.0,487.1,487.8,488,488.0,488.01,488.02,488.09,488.1,488.11,488.12,488.19,488.81 ,488.82 ,488.89 ,490,491.0,491.1,491.2,491.20,491.21,491.22,491.8,491.9,494,494.0,494.1,510.0,510.9,513.0,513.1,517.1,519.2,519.3,522.4,522.5,522.6,522.7,523.10 ,523.11 ,523.3,523.30 ,523.31 ,523.32 ,523.33 ,523.4,523.40,523.41,523.42,523.5,527.3,528.3,558.9,566,567.0,567.1,567.2,567.21,567.22,567.23,567.29,567.31,567.38,567.39,567.81,567.89,567.9,569.5,572.0,572.1,573.1,573.2,574.11,574.30,574.40,575.0,590.2,590.81,590.9,595.0,595.89,597.0,598.00,599.0,601.0,601.2,603.1,604.0,611.0,614.0,614.1,614.2,614.3,614.4,614.5,614.6,614.7,614.8,614.9,615.0,615.1,615.9,616.0,616.10,616.11,616.2,616.3,616.4,634.00,634.01,634.02,635.00,635.01,635.02,636.00,636.01,636.02,637.00,637.01,637.02,638.0,639.0,646.60,646.61,646.62,646.63,646.64,647.00 ,647.01 ,647.02 ,647.03 ,647.04 ,647.10 ,647.11,647.12 ,647.13,647.14 ,647.20 ,647.21 ,647.22 ,647.23 ,647.24 ,647.30,647.31,647.32,647.33,647.34,647.40,647.41,647.42,647.43,647.44,647.50,647.51,647.52,647.53,647.54,647.60,647.61,647.62,647.63,647.64,647.80,647.81,647.82,647.83,647.84,647.90,647.91,647.92,647.93,647.94,658.40,658.41,658.43,659.30,659.31,659.33,670.00,670.02,670.04,670.10,670.12,670.14,670.20,670.22,670.24,670.30,670.32,670.34,670.80,670.82,670.84,675.00,675.01,675.02,675.03,675.04,675.10,675.11,675.12,675.13,675.14,675.20,675.21,675.22,675.23,675.24,675.80,675.81,675.82,675.83,675.84,675.90,675.91,675.92,675.93,675.94,680.0,680.1,680.2,680.3,680.4,680.5,680.6,680.7,680.8,680.9,681.00,681.01,681.02,681.10,681.11,681.9,682.0,682.1,682.2,682.3,682.4,682.5,682.6,682.7,682.8,682.9,683,684,685.0,685.1,686.0,686.00,686.01,686.09,686.1,686.8,686.9,690.8,694.0,694.1,694.2,694.3,695.3,695.81 ,696.3,711.00,711.01,711.02,711.03,711.04,711.05,711.06,711.07,711.08,711.09,711.30,711.31,711.32,711.33,711.34,711.35,711.36,711.37,711.38,711.39,711.40,711.41,711.42,711.43,711.44,711.45,711.46,711.47,711.48,711.49,711.50,711.51,711.52,711.53,711.54,711.55,711.56,711.57,711.58,711.59,711.60,711.61,711.62,711.63,711.64,711.65,711.66,711.67,711.68,711.69,711.70,711.71,711.72,711.73,711.74,711.75,711.76,711.77,711.78,711.79,711.80,711.81,711.82,711.83,711.84,711.85,711.86,711.87,711.88,711.89,711.90,711.91,711.92,711.93,711.94,711.95,711.96,711.97,711.98,711.99,728.0,728.86,730.00,730.01,730.02,730.03,730.04,730.05,730.06,730.07,730.08,730.09,730.10,730.11,730.12,730.13,730.14,730.15,730.16,730.17,730.18,730.19,730.20,730.21,730.22,730.23,730.24,730.25,730.26,730.27,730.28,730.29,730.80,730.81,730.82,730.83,730.84,730.85,730.86,730.87,730.88,730.89,730.90,730.91,730.92,730.93,730.94,730.95,730.96,730.97,730.98,730.99,760.2,762.7,770.0,771.0,771.1,771.2,771.3,771.4,771.5,771.6,771.7,771.8,771.81,771.82,771.83,771.89,785.52,790.7,790.8,795.05,795.15,795.19,795.3,795.31,795.39,795.71,795.8,796.75,796.79,910.1,910.3,910.5,910.7,910.9,911.1,911.3,911.5,911.7,911.9,912.1,912.3,912.5,912.7,912.9,913.1,913.3,913.5,913.7,913.9,914.1,914.3,914.5,914.7,914.9,915.1,915.3,915.5,915.7,915.9,916.1,916.3,916.5,916.7,916.9,917.1,917.3,917.5,917.7,917.9,919.1,919.3,919.5,919.7,919.9,958.3,996.60,996.61,996.62,996.63,996.64,996.65,996.66,996.67,996.68,996.69,997.09,997.62,998.51,998.59,999.3,999.31,999.32,999.33,999.34,999.39 | A00.0,A00.1,A00.9,A01.0,A01.1,A01.2,A01.3,A01.4,A02.0,A02.1,A02.2+,A02.8,A02.9,A03.0,A03.1,A03.2,A03.3,A03.8,A03.9,A04.0,A04.1,A04.2,A04.3,A04.4,A04.5,A04.6,A04.7,A04.8,A04.9,A05.0,A05.1,A05.2,A05.3,A05.4,A05.8,A05.9,A06.0,A06.1,A06.2,A06.3,A06.4,A06.5+,A06.6+,A06.7,A06.8,A06.9,A07.0,A07.1,A07.2,A07.3,A07.8,A07.9,A08.0,A08.1,A08.2,A08.3,A08.4,A08.5,A09,A09.0,A09.9,A15.0,A15.1,A15.2,A15.3,A15.4,A15.5,A15.6,A15.7,A15.8,A15.9,A16.0,A16.1,A16.2,A16.3,A16.4,A16.5,A16.7,A16.8,A16.9,A17.0+,A17.1+,A17.8+,A17.9+,A18.0+,A18.1,A18.1+,A18.2,A18.3+,A18.4,A18.5+,A18.6+,A18.7+,A18.8+,A19.0,A19.1,A19.2,A19.8,A19.9,A20.0,A20.1,A20.2,A20.3,A20.7,A20.8,A20.9,A21.0,A21.1,A21.2,A21.3,A21.7,A21.8,A21.9,A22.0,A22.1,A22.2,A22.7,A22.8,A22.9,A23.0,A23.1,A23.2,A23.3,A23.8,A23.9,A24.0,A24.1,A24.2,A24.3,A24.4,A25.0,A25.1,A25.9,A26.0,A26.7,A26.8,A26.9,A27.0,A27.8,A27.9,A28.0,A28.1,A28.2,A28.8,A28.9,A30.0,A30.1,A30.2,A30.3,A30.4,A30.5,A30.8,A30.9,A31.0,A31.1,A31.8,A31.9,A32.0,A32.1,A32.7,A32.8,A32.9,A33,A34,A35,A36.0,A36.1,A36.2,A36.3,A36.8,A36.8+,A36.9,A37.0,A37.1,A37.8,A37.9,A38,A39.0+,A39.1+,A39.2,A39.3,A39.4,A39.5,A39.8,A39.8+Other,A39.9,A40.0,A40.1,A40.2,A40.3,A40.8,A40.9,A41.0,A41.1,A41.2,A41.3,A41.4,A41.51,A41.52,A41.58,A41.8,A41.9,A42.0,A42.1,A42.2,A42.7,A42.8,A42.9,A43.0,A43.1,A43.8,A43.9,A44.0,A44.1,A44.8,A44.9,A46,A48.0,A48.1,A48.2,A48.3,A48.4,A48.8,A49.0,A49.1,A49.2,A49.3,A49.8,A49.9,A50.0,A50.1,A50.2,A50.3,A50.4,A50.5,A50.6,A50.7,A50.9,A51.0,A51.1,A51.2,A51.3,A51.3+,A51.4,A51.5,A51.9,A52.0+,A52.1,A52.1+,A52.2,A52.3,A52.7,A52.7+,A52.8,A52.9,A53.0,A53.9,A54.0,A54.1,A54.2+,A54.3,A54.3+,A54.4+,A54.5,A54.6,A54.8,A54.8+,A54.9,A55,A56.0,A56.1,A56.2,A56.3,A56.4,A56.8,A57,A58,A59.0,A59.0+,A59.8,A59.9,A60.0,A60.1,A60.9,A63.0,A63.8,A64,A65,A66.0,A66.1,A66.2,A66.3,A66.4,A66.5,A66.6,A66.7,A66.8,A66.9,A67.0,A67.1,A67.2,A67.3,A67.9,A68.0,A68.1,A68.9,A69.0,A69.1,A69.2,A69.8,A69.9,A70,A70+,A71.0,A71.1,A71.9,A74.0+,A74.8,A74.8+,A74.9,A75.0,A75.1,A75.2,A75.3,A75.9,A77.0,A77.1,A77.2,A77.3,A77.8,A77.9,A78,A79.0,A79.1,A79.8,A79.9,A80.1,A80.2,A80.3,A80.4,A80.9,A81.0,A81.1,A81.2,A81.8,A81.9,A82.0,A82.1,A82.9,A83.0,A83.1,A83.2,A83.3,A83.4,A83.5,A83.6,A83.8,A83.9,A84.0,A84.1,A84.8,A84.9,A85.0,A85.1,A85.2,A85.8,A86,A87.0+,A87.1+,A87.2,A87.8,A87.9,A88.0,A88.8,A89,A90,A91,A92.0,A92.1,A92.2,A92.3,A92.4,A92.8,A92.9,A93.0,A93.1,A93.2,A93.8,A94,A95.0,A95.1,A95.9,A96.0,A96.1,A96.2,A96.8,A96.9,A98.0,A98.1,A98.2,A98.3,A98.4,A98.5,A98.8,A99,B00.0,B00.1,B00.2,B00.3+,B00.4+,B00.5+,B00.7,B00.8,B00.9,B01.0,B01.1+,B01.2+,B01.8,B01.9,B02.0,B02.1+,B02.2+,B02.3+,B02.7,B02.8,B02.9,B03,B04,B05.0+,B05.1,B05.2+,B05.3+,B05.4,B05.8,B05.9,B06.0+,B06.8,B06.9,B07,B08.0,B08.1,B08.2,B08.3,B08.4,B08.5,B08.8,B09,B15.0,B15.9,B16.0,B16.1,B16.2,B16.9,B17.0,B17.1,B17.2,B17.8,B17.9,B18.0,B18.1,B18.2,B18.8,B18.9,B19.0,B19.9,B20.0,B20.1,B20.2,B20.3,B20.4,B20.5,B20.6,B20.7,B20.8,B20.9,B21.0,B21.1,B21.2,B21.3,B21.7,B21.8,B21.9,B22.0,B22.1,B22.2,B22.7,B23.0,B23.1,B23.2,B23.8,B24,B25.0,B25.1,B25.2,B25.8,B25.9,B26.0+,B26.1+,B26.2+,B26.3+,B26.8,B26.8+,B26.9,B27.0,B27.1,B27.8,B27.9,B30.0+,B30.1+,B30.2+,B30.3+,B30.8+,B30.9,B33.0,B33.1,B33.2,B33.3,B33.4,B33.8,B34.0,B34.1,B34.2,B34.3,B34.4,B34.8,B34.9,B35.0,B35.1,B35.2,B35.3,B35.4,B35.5,B35.6,B35.8,B35.9,B36.0,B36.1,B36.2,B36.3,B36.8,B36.9,B37.0,B37.1,B37.2,B37.3+,B37.4+,B37.5+,B37.6,B37.7,B37.81,B37.88,B37.9,B38.0,B38.1,B38.2,B38.3,B38.4+,B38.7,B38.8,B38.9,B39.0,B39.1,B39.2,B39.3,B39.4,B39.5,B39.9,B40.0,B40.1,B40.2,B40.3,B40.7,B40.8,B40.9,B41.0,B41.7,B41.8,B41.9,B42.0+,B42.1,B42.7,B42.8,B42.9,B43.0,B43.1,B43.2,B43.8,B43.9,B44.0,B44.1,B44.2,B44.7,B44.8,B44.9,B45.0,B45.1,B45.2,B45.3,B45.7,B45.8,B45.9,B46.0,B46.1,B46.2,B46.3,B46.4,B46.5,B46.8,B46.9,B47.0,B47.1,B47.9,B48.0,B48.1,B48.2,B48.3,B48.4,B48.7,B48.8,B49,B50.0,B50.8,B50.9,B51.0,B51.8,B51.9,B52.0,B52.8,B52.9,B53.0,B53.1,B53.8,B54,B55.0,B55.1,B55.2,B55.9,B56.0,B56.1,B56.9,B57.0,B57.1,B57.2,B57.3,B57.4,B57.5,B58.0+,B58.1+,B58.2+,B58.3+,B58.8,B58.9,B59,B60.0,B60.1,B60.2,B60.8,B64,B65.0,B65.1,B65.2,B65.3,B65.8,B65.9,B66.0,B66.1,B66.2,B66.3,B66.4,B66.5,B66.8,B66.9,B67.0,B67.1,B67.2,B67.3,B67.3+,B67.4,B67.5,B67.6,B67.7,B67.8,B67.9,B68.0,B68.1,B68.9,B69.0,B69.1,B69.8,B69.9,B70.0,B70.1,B71.0,B71.1,B71.8,B71.9,B72,B73,B74.0,B74.1,B74.2,B74.3,B74.4,B74.8,B74.9,B75,B76.0,B76.1,B76.8,B76.9,B77.0,B77.8,B77.9,B78.0,B78.1,B78.7,B78.9,B79,B80,B81.0,B81.1,B81.2,B81.3,B81.4,B81.8,B82.0,B82.9,B83.0,B83.1,B83.2,B83.3,B83.4,B83.8,B83.9,B85.0,B85.1,B85.2,B85.3,B85.4,B86,B87.0,B87.1,B87.2,B87.3,B87.4,B87.8,B87.9,B88.0,B88.1,B88.2,B88.3,B88.8,B88.9,B89,B95.0,B95.1,B95.2,B95.3,B95.41,B95.42,B95.48,B95.5,B95.6,B95.7,B95.8,B96.0,B96.1,B96.2,B96.3,B96.4,B96.5,B96.6,B96.7,B96.81,B96.88,B97.0,B97.1,B97.2,B97.3,B97.4,B97.5,B97.6,B97.7,B97.8,B98.0,B98.1,B99,D59.3,G00.0,G00.1,G00.1*,G00.2,G00.3,G00.8,G00.9,G01,G02.0*,G02.1*,G02.8*,G03.0,G03.1,G03.2,G03.8,G03.9,G04.0,G04.1,G04.2,G04.8,G04.9,G05.0,G05.1*,G05.2*,G05.8,G06.0,G06.1,G06.2,G07,G08,G92,G94.0,H00.0,H00.1,H01.0,H01.8,H03.0*,H03.1*,H04.0,H06.1*,H10.0,H10.2,H10.3,H10.5,H13.0,H13.1*,H19.0,H19.1,H19.2*,H20.0,H22.0,H32.0,H44.0,H44.1,H45.1,H48.1,H58.8,H60.0,H60.1,H60.2,H60.3,H62.0,H62.1,H62.2*,H62.3,H65.0,H65.1,H65.2,H65.3,H65.4,H65.9,H66.0,H66.1,H66.2,H66.3,H66.4,H66.9,H67.0,H67.1,H68.0,H70.0,H70.1,H70.2,H70.8,H70.9,H72.0,H72.1,H72.2,H72.8,H72.9,H73.0,H75.0,H83.0,H92.1,H94.0,I00,I01.0,I01.1,I01.2,I01.8,I01.9,I30.1,I32.0,I32.1,I33.0,I39.0,I39.1,I39.2,I39.3,I39.4,I39.8,I40.0,I41.0,I41.1,I41.2,I43.0,I52.0,I52.1,I68.1,I98.0,I98.1,J00,J01.0,J01.1,J01.2,J01.3,J01.4,J01.8,J01.9,J02.0,J02.8,J02.9,J03.0,J03.8,J03.9,J04.0,J04.1,J04.2,J05.0,J05.1,J06.0,J06.8,J06.9,J09,J10.0,J10.1,J10.8,J11,J11.0,J11.1,J11.8,J12.0,J12.1,J12.2,J12.3,J12.8,J12.9,J13,J14,J15.0,J15.1,J15.2,J15.3,J15.4,J15.5,J15.6,J15.7,J15.8,J15.9,J16.0,J16.8,J17.0*,J17.1*,J17.2*,J17.3,J17.8*,J18.0,J18.1,J18.8,J18.9,J20.0,J20.1,J20.2,J20.3,J20.4,J20.5,J20.6,J20.7,J20.8,J20.9,J21.0,J21.1,J21.8,J21.9,J22,J32.0,J32.1,J32.2,J32.3,J32.4,J32.8,J32.9,J34.0,J35.0,J36,J39.0,J39.1,J39.2,J40,J41.0,J41.1,J41.8,J42,J44.0,J47,J65,J85.0,J85.1,J85.2,J85.3,J86.0,J86.9,J99.8,K04.4,K04.5,K04.6,K04.7,K05.2,K05.3,K05.4,K11.3,K12.2,K52.8,K61.0,K61.1,K61.2,K61.3,K61.4,K63.0,K65.0,K65.8,K65.9,K67.0,K67.1,K67.2,K67.3,K67.8*,K75.0,K75.1,K77.0*,K81.0,K87.1,K90.8+,K93.0,K93.1,L00,L01.0,L01.1,L02.0,L02.1,L02.2,L02.3,L02.4,L02.8,L02.9,L03.0,L03.01,L03.02,L03.1,L03.10,L03.11,L03.2,L03.3,L03.8,L03.9,L04.0,L04.1,L04.2,L04.3,L04.8,L04.9,L05.0,L05.9,L08.0,L08.1,L08.8,L08.9,L13.0,L30.3,L54.0,L88,M00.0,M00.1,M00.2,M00.8,M00.90,M00.91,M00.92,M00.93,M00.94,M00.95,M00.96,M00.97,M00.98,M00.99,M01.0,M01.1,M01.2,M01.30*,M01.31*,M01.32*,M01.33*,M01.34*,M01.35*,M01.36*,M01.37*,M01.38*,M01.39*,M01.4,M01.50*,M01.51*,M01.52*,M01.53*,M01.54*,M01.55*,M01.56*,M01.57*,M01.58*,M01.59*,M01.60*,M01.61*,M01.62*,M01.63*,M01.64*,M01.65*,M01.66,M01.67*,M01.68,M01.69*,M01.80*,M01.81*,M01.82*,M01.83,M01.83*,M01.84*,M01.85*,M01.86*,M01.87*,M01.88*,M01.89*,M46.2,M46.3,M46.4,M46.5,M49.0,M49.1,M49.2,M49.3,M60.0,M60.09,M63.0,M63.1,M63.2,M65.0,M65.1,M68.0,M71.0,M71.1,M72.6,M72.8,M73.0,M73.1,M86.0,M86.10,M86.11,M86.12,M86.13,M86.14,M86.15,M86.16,M86.17,M86.18,M86.19,M86.2,M86.4,M86.5,M86.60,M86.67,M86.68,M86.69,M86.8,M86.90,M86.91,M86.92,M86.93,M86.94,M86.95,M86.96,M86.97,M86.98,M86.99,M90.0,M90.1,N08.0,N08.8,N13.6,N15.1,N15.9,N16.0,N22.0,N29.1,N30.0,N30.8,N33.0,N33.8,N34.0,N35.1,N39.0,N41.0,N41.2,N43.1,N45.0,N45.9,N51.0,N51.1,N51.2,N51.8,N61,N70.0,N73.0,N73.1,N73.2,N73.3,N73.4,N73.5,N73.6,N73.8,N73.9,N74.0,N74.1,N74.2,N74.3,N74.4,N74.8,N75.0,N75.1,N76.0,N76.4,N76.6,N76.8,N77.0,N77.1*,O03.0,O03.5,O04.0,O04.5,O05.0,O05.5,O06.0,O06.5,O07.0,O07.5,O08.0,O23.0,O23.1,O23.2,O23.3,O23.4,O23.5,O23.9,O26.4,O41.1,O75.3,O85,O86.0,O86.1,O86.2,O86.3,O86.4,O86.8,O91.00,O91.10,O91.20,O98.0,O98.1,O98.2,O98.3,O98.4,O98.5,O98.6,O98.7,O98.8,O98.9,P00.2,P02.7,P23.0,P23.1,P23.2,P23.3,P23.4,P23.5,P23.6,P23.8,P23.9,P35.0,P35.1,P35.2,P35.3,P35.8,P35.9,P36.0,P36.1,P36.2,P36.3,P36.4,P36.5,P36.8,P36.9,P37.0,P37.1,P37.2,P37.3,P37.4,P37.5,P37.8,P37.9,P38,P39.0,P39.1,P39.2,P39.3,P39.4,P39.8,P39.9,V08,V09.0,V09.1,V09.2,V09.3,V09.4,V09.50,V09.51,V09.6,V09.70,V09.71,V09.80,V09.81,V09.90,V09.91,Z21 |
