# Supplementary material for: Childhood Hospitalisation with Infection and Cardiovascular Disease in Early-Mid Adulthood: A Longitudinal Population-Based Study
Source: PLoS One. 2015 May 4;10(5):e0125342. doi: 10.1371/journal.pone.0125342 (PMC4418819; doi:10.1371/journal.pone.0125342)
Supplement: S3 Table — (DOCX) [file pone.0125342.s003.docx]

**TABLE S3:** Adjusted Hazard Ratios^1^ for CVD Hospitalisation in Adulthood in Relation to Number of Infection-Related Childhood Hospitalisations on Subjects with Birth Weight Data

|  | **% [N]** | |  |
| --- | --- | --- | --- |
|  | **Cases** | **Controls** | **Adjusted Hazard Ratio (CI)** |
| **Count** |  |  |  |
| 0 | 33.8 [23] | 58.3. [430] | 1 |
| 1 | 22.1 [15] | 22.5 [166] | 1.3 (0.7-2.6) |
| 2 | 8.8. [6] | 9.2 [68] | 0.6 (0.2-2.2) |
| 3+ | 35.3. [25] | 10.0 [74] | 2.3 (1.1-5.0) ^*^ |

^1^ Hazard Ratios are adjusted for year of birth, sex, Indigenous status, POBW and social disadvantage

^*^ P=0.038
