# Supplementary material for: Childhood Hospitalisation with Infection and Cardiovascular Disease in Early-Mid Adulthood: A Longitudinal Population-Based Study
Source: PLoS One. 2015 May 4;10(5):e0125342. doi: 10.1371/journal.pone.0125342 (PMC4418819; doi:10.1371/journal.pone.0125342)
Supplement: S4 Table — (DOCX) [file pone.0125342.s004.docx]

**TABLE S4:** ICD9 and ICD10 Procedure Codes for chronic or recurrent middle ear infections

| **Subset** | **ICD9** | **ICD10** |
| --- | --- | --- |
| Ear infections | 19.4, 19.52, 19.53, 19.54, 19.55, 19.6, 20.01, 20.09,  20.1, 193 | 41527-00, 41530-00, 41533-01, 41536-01, 41542-00, 41626-00, 41626-01, 41632-00, 41632-00, 41632-01, 41632-01, 41635-01,  41638-01, 41644-00 |
